# Fatal autonomic failure due to premanifesting Parkinson's disease only diagnosed at autopsy

**DOI:** 10.1002/ccr3.3042

**Published:** 2020-06-30

**Authors:** Istvan Bodi, Ellen Merete Hagen, Gordon Thorpe Ingle, Michael P Lunn

**Affiliations:** ^1^ Clinical Neuropathology King's College Hospital NHS Foundation Trust London UK; ^2^ Autonomic Unit National Hospital for Neurology and Neurosurgery London UK; ^3^ Neurology Department Whipps Cross University Hospital London UK; ^4^ Centre for Neuromuscular Disease National Hospital for Neurology and Neurosurgery London UK

**Keywords:** autonomic dysfunction, Lewy body disease, multiple system atrophy, neurodegeneration, Parkinson's disease, α‐synuclein

## Abstract

A 46‐year‐old male had 11‐year history of cryptic autonomic dysfunction. He developed a fatal autonomic failure with diffuse hypoxic brain injury. Histology examination of medulla oblongata and the celiac ganglion revealed many α‐synuclein immunoreactive Lewy bodies confirming the diagnosis of premanifesting Parkinson's disease (PD). PNS involvement in PD is underappreciated.

## Graphical abstract

Parkinson's disease affects the peripheral autonomic system and may cause autonomic dysfunction without typical parkinsonian symptoms. This case also underlines the importance of histological sampling of the PNS in cases with autonomic dysfunction.

## 1 INTRODUCTION

Peripheral nervous system involvement in Parkinson's disease (PD) is not frequently identified despite autonomic dysfunction having been recognized since the original description by James Parkinson in 1817. The autonomic dysfunction is usually central or drug related, but it is recognized that peripheral autonomic dysfunction sometime contributes. Symptoms of dysautonomia are variable and can be cardiovascular, gastrointestinal, urogenital, sudomotor and thermoregulatory, and pupillary, accompanied by sleep and respiratory disorders.^1^, ^2^, ^3^ Rarely, PD may present with autonomic failure.^4^ We present a case with fatal autonomic failure due to premanifesting PD diagnosed at autopsy.

## 2 CASE REPORT

A 46‐year‐old man had an out of hospital cardiac arrest after complaining of dizziness and received delayed bystander CPR. He was admitted to ITU where he was found to have severe hypoxic‐ischemic encephalopathy with GCS of 3‐4. Despite maximal medical therapy over an extended admission, GCS did not improve and he eventually developed hospital‐acquired pneumonia and died 15 weeks after admission. No cause for the cardiac arrest was identified despite extensive investigation. HM Coroner ordered a postmortem examination.

The patient first presented to cardiology in March 2011 with a 4‐year history of episodes of positional dizziness and occasional collapses. He fainted upon changing position, that is, sitting up and going upstairs. He had lost the ability to sweat when playing tennis and developed erectile dysfunction. There was no family history of neurological disease.

He was referred to a neurologist with diagnosis of “autonomic dysfunction” and was followed in the Autonomic Unit in the National Hospital for Neurology & Neurosurgery for 7 years, during which he was seen by four different consultants. A year after presentation, he had coat‐hanger pain, light headedness on standing, poor heat tolerance, erectile dysfunction, bouts of sweating, dry mouth, and episodic syncopal collapses. On examination, there were normal eye movements and no pyramidal, extrapyramidal, or cerebellar symptoms. Cardiovascular autonomic tests in March 2013 showed marked symptomatic orthostatic hypotension on head up tilt and standing. There was no pressor responsiveness, the Valsalva maneuver was blocked, and there was no heart rate variability. Overall, there was evidence for severe cardiovascular autonomic failure. The basal catecholamine levels were slightly low, with a minimal rise on postural change. Investigations did not reveal any evidence of multiple system atrophy or other neurodegenerative processes. He was advised to hydrate and make use of calf exercise before getting up from bed, use compression stockings during the day, eat frequent small meals, add salt to the food, and do regular exercise.

Two years after symptom presentation, neurological examination found decreased vibration sensation in ankles with intact proprioception. Clinical neurophysiological investigation suggested a length‐dependent sensorimotor axonal polyneuropathy. Blood tests were negative for syphilis, HIV, celiac serology, ANA, ENA, ANCA, dsDNA, thyroid profile, CRP, antineuronal antibodies, complement, fasting glucose, and antibodies to preganglionic nicotinic acetylcholine receptors. A fat biopsy was negative for amyloid, and genetic testing was negative for hereditary sensory motor neuropathies.

Five years after presentation, he changed his job to night shifts to avoid the heat during summer. If he became angry at his dog, he felt shattered and needed to lie down, consistent with a lack of adrenaline response. He was due for annual checkup but sadly had a cardiac arrest and developed diffuse hypoxic brain damage.

**FIGURE 1:**
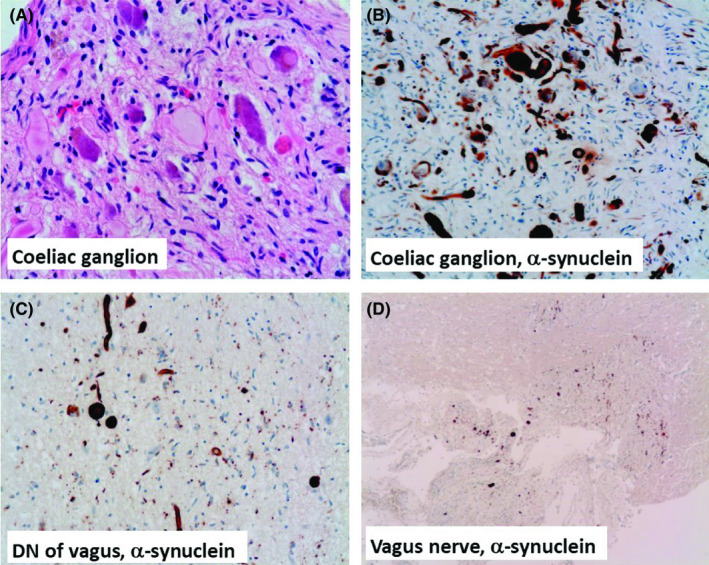
Postmortem histology. A, The celiac ganglion reveals typical Lewy bodies and thick axonal hyalinous structures in a background of neuronal loss. B, Immunohistochemistry demonstrates many α‐synuclein positive cytoplasmic and numerous axonal structures. The medulla oblongata also shows also many α‐synuclein immunoreactive structures in the dorsal nucleus (DN) of vagus (C) and the vagus nerve itself (D)

Autopsy examination revealed bronchopneumonia and bilateral pyelonephritis, confirmed by histology. There was no obvious pathology in the heart. A possibility of Addison's disease was considered, but the adrenal glands did not show atrophy. According to local Coroner's practice, histology was limited to 10 blocks and carried out on the following organs: heart, lung, kidney, adrenal gland, pituitary, celiac ganglion, superior frontal gyrus, hippocampus, cerebellum, and medulla oblongata. Histology examination of the brain confirmed diffuse hypoxic brain injury in keeping with 15 weeks of survival. The medulla oblongata, particularly the dorsal nucleus of the vagus, and the celiac ganglion revealed many Lewy bodies and numerous thick dystrophic neurites associated with neuronal loss (Figure 1). Immunohistochemistry for α‐synuclein confirmed presence of numerous Lewy bodies and thick dystrophic neurites in similar locations (Figure 1). No α‐synuclein pathology was seen in the frontal neocortex and the hippocampus. The substantia nigra was well pigmented by naked eye, therefore, not sampled due to restriction of number of blocks taken. The overall features were in keeping with the diagnosis of brainstem‐predominant Lewy body disease, equivalent of premanifesting PD, with predominant involvement of the peripheral autonomic system.

## 3 DISCUSSION

This patient had a long history of cryptic autonomic dysfunction. He was extensively investigated, but no cause had been found. There was no clinical sign of a central neurodegenerative disease, and he did not reveal parkinsonian symptoms on examination. Unfortunately, he developed a catastrophic autonomic failure leading to cardiac arrest and subsequent diffuse hypoxic brain injury. Neuropathological examination confirmed that the autonomic failure was almost certainly secondary to Lewy body pathology involving the autonomic ganglia and the medulla oblongata, consistent with premanifesting PD. There were also numerous α‐synuclein immunoreactive dystrophic axons in the vagus nerve and the celiac ganglion. Interestingly, the substantia nigra was well pigmented by naked eye indicating no significant neuronal loss at that site explaining the lack of obvious parkinsonian symptoms.

It is well recognized that Parkinson's disease affects the peripheral autonomic system and may cause autonomic dysfunction.^5^, ^6^ It has been demonstrated that hyperphosphorylated α‐synuclein deposition can be detected in various peripheral organs (eg, gastrointestinal tract, autonomic ganglia, submandibular gland, and skin) in PD.^7^, ^8^, ^9^, ^10^, ^11^, ^12^ Autonomic failure has been also recognized in Parkinson's disease, but it is rare and not considered a fatal complication. In this particular case, however, the involvement of the peripheral autonomic system resulted in autonomic failure and subsequent cardiac arrest leading to diffuse hypoxic brain damage. It is also recorded in the literature that the Lewy body pathology (α‐synucleinopathy) also affects the peripheral autonomic system and the autonomic dysfunction may proceed development of typical parkinsonian symptoms, eventually progressing to either Parkinson's disease or diffuse Lewy body disease.^4^, ^13^ In our knowledge, no fatal autonomic failure due to PD has been published in the literature. This case also underlines the importance of histological sampling of the peripheral nervous system in cases with autonomic dysfunction and also in suspected PD.

## 4 CONSENT

Written consent was obtained from the next of kin for this publication.

## CONFLICT OF INTEREST

The authors declare that they have no conflict of interest.

## AUTHOR CONTRIBUTIONS

IB: performed the autopsy, made the neuropathological diagnosis, and generated the images. EMH, GTI, and ML: treated and followed the patient and revised the manuscript. IB: reviewed the literature and drafted the manuscript. All authors approved the final version of this manuscript.

## References

[1] Chaudhuri KR . Autonomic dysfunction in movement disorders. Curr Opin Neurol. 2001;14(4):505‐511. 11470968 10.1097/00019052-200108000-00012

[2] Siddiqui MF , Rast S , Lynn MJ , Auchus AP , Pfeiffer RF . Autonomic dysfunction in Parkinson's disease: a comprehensive symptom survey. Park Relat Disord. 2002;8(4):277‐284. 10.1016/s1353-8020(01)00052-9 12039423

[3] De Pablo‐Fernandez E , Tur C , Revesz T , et al. Association of autonomic dysfunction with disease progression and survival in Parkinson disease. JAMA Neurol. 2017;74(8):970‐976. 28655059 10.1001/jamaneurol.2017.1125 PMC5710320

[4] Singer W , Berini SE , Sandroni P , et al. Pure autonomic failure: predictors of conversion to clinical CNS involvement. Neurology. 2017;88(12):1129‐1136. 28202694 10.1212/WNL.0000000000003737 PMC5373781

[5] Jain S . Multi‐organ autonomic dysfunction in Parkinson disease. Park Relat Disord. 2011;17(2):77‐83. 10.1016/j.parkreldis.2010.08.022 PMC3021587 20851033

[6] Kaufmann H , Goldstein DS . Autonomic dysfunction in Parkinson disease. Handb Clin Neurol. 2013;117:259‐278. 24095131 10.1016/B978-0-444-53491-0.00021-3

[7] Braak H , De Vos RAI , Bohl J , Del Tredici K . Gastric α‐synuclein immunoreactive inclusions in Meissner's and Auerbach's plexuses in cases staged for Parkinson's disease‐related brain pathology. Neurosci Lett. 2006;396(1):67‐72. 16330147 10.1016/j.neulet.2005.11.012

[8] Lee HJ , Jung KW , Chung SJ , et al. Relation of enteric α‐synuclein to gastrointestinal dysfunction in patients with parkinson's disease and in neurologically intact subjects. J Neurogastroenterol Motil. 2018;24(3):469‐478. 29969861 10.5056/jnm17141 PMC6034677

[9] Yan F , Chen Y , Li M , et al. Gastrointestinal nervous system a‐synuclein as a potential biomarker of Parkinson disease. Med (United States). 2018;97(28):1‐8. 10.1097/MD.0000000000011337 PMC6076112 29995769

[10] Wang N , Gibbons CH , Freeman R . Cutaneous α‐synuclein and Parkinson disease, a biomarker of disease severity. Auton Neurosci. 2013;177(1):27‐28.

[11] Sariahmetoglu H , Soysal A , Sen A , et al. Forehead sympathetic skin responses in determining autonomic involvement in Parkinson's disease. Clin Neurophysiol. 2014;125(12):2436‐2440. 24780107 10.1016/j.clinph.2014.03.024

[12] Shin J , Park SH , Shin C , et al. Submandibular gland is a suitable site for alpha synuclein pathology in Parkinson disease. Park Relat Disord. 2019;58:35‐39. 10.1016/j.parkreldis.2018.04.019 30340912

[13] Kaufmann H , Nahm K , Purohit D , Wolfe D . Autonomic failure as the initial presentation of Parkinson disease and dementia with Lewy bodies. Neurology. 2004;63(6):1093‐1095. 15452307 10.1212/01.wnl.0000138500.73671.dc

